# Supplementary material for: Conditional chemoconnectomics (cCCTomics) as a strategy for efficient and conditional targeting of chemical transmission
Source: eLife. 2024 Apr 30;12:RP91927. doi: 10.7554/eLife.91927 (PMC11060718; doi:10.7554/eLife.91927)
Supplement: Figure 3—source data 3. [file elife-91927-fig3-data3.zip › Figure 3 source data3 part 2.zip!/Figure 3-Source data 3-part2/1060f2-1-CRISPResso_Report_gl5umwyq.zip!/CRISPResso_on_1060f2_4/1c.Alignment_barplot.pdf]

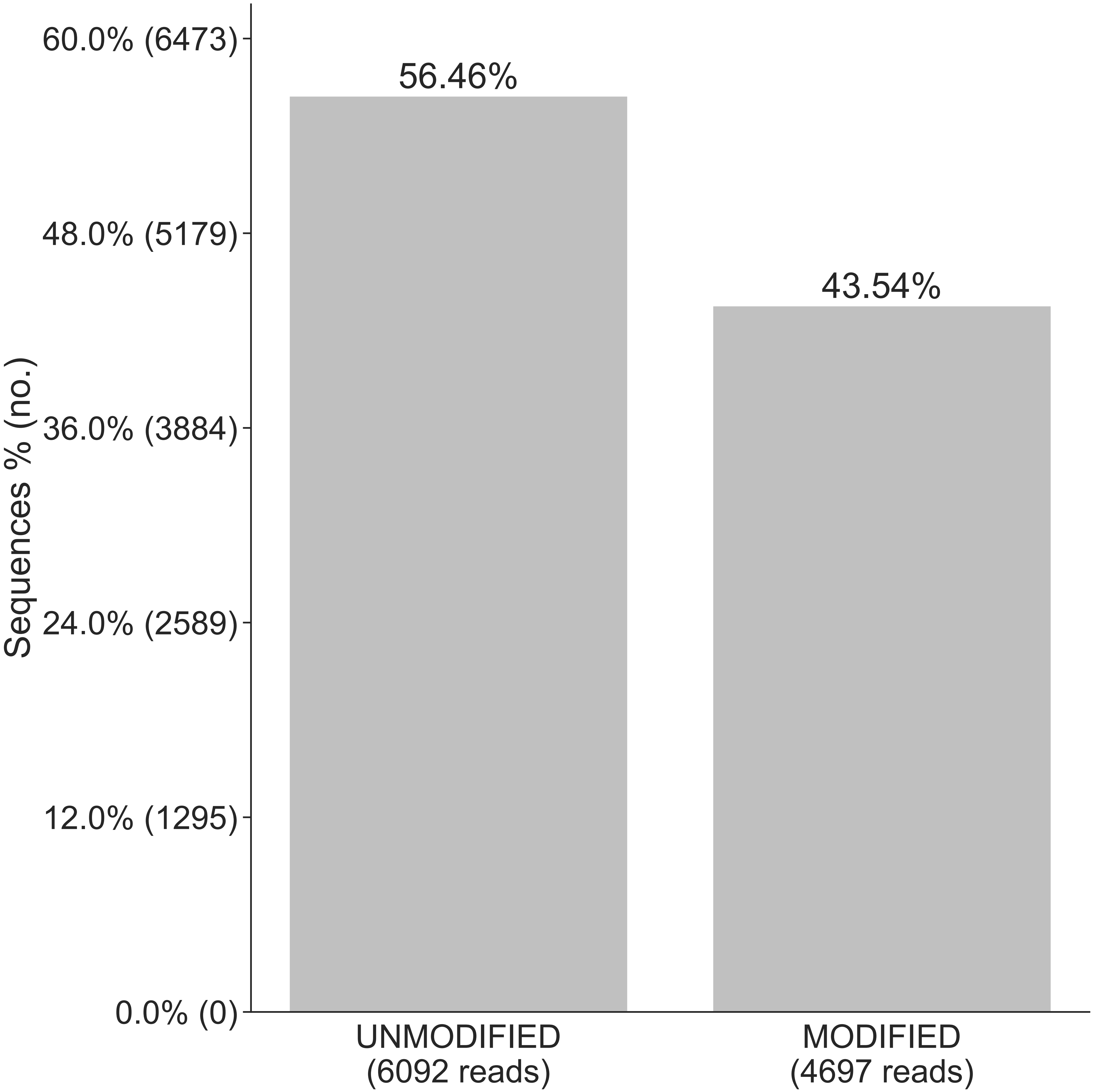

Sequences % (no.)
60.0% (6473)
48.0% (5179)
36.0% (3884)
24.0% (2589)
12.0% (1295)
0.0% (0)
56.46%
43.54%
UNMODIFIED
(6092 reads)
MODIFIED
(4697 reads)
